# Proanthocyanidins against Oxidative Stress: From Molecular Mechanisms to Clinical Applications

**DOI:** 10.1155/2018/8584136

**Published:** 2018-03-12

**Authors:** Lingyu Yang, Dehai Xian, Xia Xiong, Rui Lai, Jing Song, Jianqiao Zhong

**Affiliations:** ^1^Department of Dermatology, The Affiliated Hospital of Southwest Medical University, Luzhou 646000, China; ^2^Department of Anatomy, Southwest Medical University, Luzhou 646000, China

## Abstract

Proanthocyanidins (PCs) are naturally occurring polyphenolic compounds abundant in many vegetables, plant skins (rind/bark), seeds, flowers, fruits, and nuts. Numerous* in vitro* and* in vivo* studies have demonstrated myriad effects potentially beneficial to human health, such as antioxidation, anti-inflammation, immunomodulation, DNA repair, and antitumor activity. Accumulation of prooxidants such as reactive oxygen species (ROS) exceeding cellular antioxidant capacity results in oxidative stress (OS), which can damage macromolecules (DNA, lipids, and proteins), organelles (membranes and mitochondria), and whole tissues. OS is implicated in the pathogenesis and exacerbation of many cardiovascular, neurodegenerative, dermatological, and metabolic diseases, both through direct molecular damage and secondary activation of stress-associated signaling pathways. PCs are promising natural agents to safely prevent acute damage and control chronic diseases at relatively low cost. In this review, we summarize the molecules and signaling pathways involved in OS and the corresponding therapeutic mechanisms of PCs.

## 1. Introduction

For centuries, natural plant extracts have been used to prevent and treat a variety of clinical diseases. Proanthocyanidins (PCs) are ubiquitous in fruits, seeds, cereals, bark, flowers, nuts, and vegetables. PCs function as powerful scavengers of oxygen free radicals, with potency comparable to vitamins C and E [1, 2]. In addition, emerging evidence indicates that PCs target deleterious signaling pathways activated downstream of free radical production.

Under normal physiological conditions, the endogenous antioxidative system maintains a dynamic redox equilibrium, which is vital to many of the molecular cascades contributing to differentiation, metabolism, proliferation, and apoptosis [3]. However, excessive generation of reactive nitrogen species (RNS) and reactive oxygen species (ROS) with ensuing hyperactivation of redox-regulated protein signaling networks induce oxidative stress (OS), which is a central self-sustaining pathogenic process in various diseases of otherwise distinct etiology [4]. During disease progression, various inflammatory mediators and cytokines [e.g., nitric oxide (NO), prostaglandins, tumor necrosis factor-*α* (TNF-*α*), interleukin (IL)-1, and IL-6] produced by macrophages, neutrophils, and lymphocytes can exacerbate OS [5]. OS damages proteins, DNA, and lipids, ultimately resulting in tissue dysfunction [6]. Indeed, many neurodegenerative disorders, inflammatory diseases, cardiovascular diseases, and metabolic disorders involve OS [7–11].

Several lines of evidence have shown that natural dietary antioxidants are effective for disease prevention and treatment. Some of these antioxidants are direct free radical scavengers, whereas others such as PCs can attenuate OS both by scavenging free radicals and by modifying signaling pathways, including those involving nuclear factor erythroid 2-related factor 2 (Nrf2), mitogen-activated protein kinase (MAPK), nuclear factor-kappaB (NF-*κ*B), and phosphoinositide 3-kinase (PI3K)/Akt [12]. The antioxidant efficacy of PCs has been verified in human, animal, and culture studies, which collectively demonstrate the potential of PCs for prevention or treatment of OS-associated diseases [13–15]. Further, PCs are easily extracted, are affordable, and demonstrate low toxicity [16]. Identification of molecular events and signaling pathways involved in the antioxidant mechanism of specific PCs is crucial for further clinical applications.

## 2. Structure, Distribution, and Chemical Characteristics of PCs

### 2.1. Stereochemical Structure and Forms

**Figure 1:**
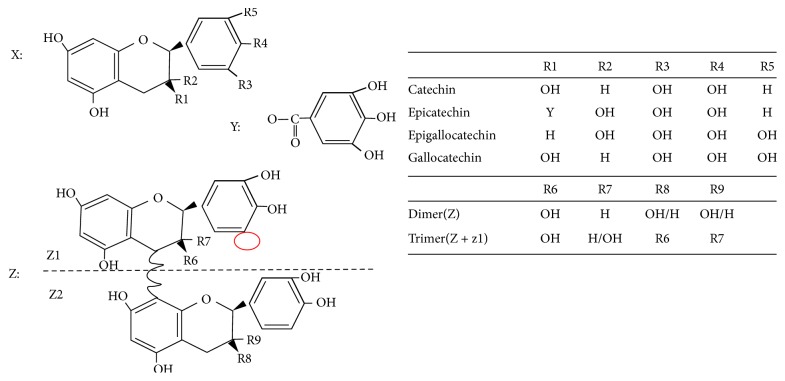
*The stereochemical structure and forms of PCs*. X represents the universal structure of flavan-3-ol units in PCs; Y indicates that the subunit is part of a constituent monomer; Z represents the basic structure of OPCs and PPCs. Z1 combines repeatedly with Z2 to form polymeric PCs.

PCs consist of chains of epicatechin, catechin, gallocatechin, or epigallocatechin subunits doubly linked through C4–C6 and C4–C8 interflavanoid bonds [17–19]. PCs that are consisting mainly of epicatechin monomers, called procyanidins, are the most abundant form. Less common PCs containing epigallocatechin subunits are designated prodelphinidins [20]. Usually, PCs with a lower degree of polymerization (two to four monomers) are named oligomeric PCs (OPCs), whereas those with more than five monomers are called polymeric PCs (PPCs) [21]. In addition, eight distinct structures of PCs dimers and trimers have been identified (B1−B8), which are designated C*n* (where *n* = 1,2,…) according to the different component monomers and positions of the connected carbon atoms [22, 23].  Figure 1 illustrates the stereochemical structure and different forms of PCs.

### 2.2. Distribution in Plants

In 1967, Joslyn et al. isolated four polyphenolic compounds from* grape* peel and seed extracts and found that these compounds could produce anthocyanidins through the cleavage of interflavan bonds using acidic butanol solution (n-BuOH/HCl, 95 : 5) in the presence of iron (III) salts and heat (95°C), which are called PCs [24, 25]. Many subsequent studies were conducted to investigate the distribution and functions of these and additional PCs. Afterwards, PCs with distinct structures and subunit compositions have been isolated in different relative proportions from numerous fruits, seeds, peels, leaves, flowers, roots, and stems (Table 1) [26], including common dietary species such as* grapes*,* apples*,* lychee*,* blackberry*, and* blueberry*.

### 2.3. Metabolism, Features, and Functions

PCs monomers are extensively conjugated in the liver and then released to either circulate in the body before excretion in urine or accumulate in tissues, partly returning to the intestine via the bile [28, 29]. Dimers and some trimers may be absorbed in the small intestine [30], but most ingested PCs are extensively depolymerized and absorbed as monomers or metabolized by the gut microbiota before passing through the colon [31]. The absorptivity of a given PC depends on its specific molecular structure; thus, molecular structure markedly influences bioavailability and function. Monomeric PCs are more absorbable than dimeric PCs. Small-molecule PCs, such as catechin monomers, tend to be easily absorbed, whereas PCs with large (−)-epigallocatechin-3-gallate molecules are poorly absorbed through the gut barrier [32, 33]. Moreover, other plant constituents consumed with PCs, such as carbohydrates, proteins, and fiber, may have synergic or antagonistic effects on PCs absorption and bioavailability [34, 35]. In some instances, poor absorption can actually contribute to efficacy against infectious diseases; for example, the benefits of PCs against urinary tract infections may occur via the interactions at the gastrointestinal tract mucosal surface [36]. In most cases, however, the benefits of small-molecule PCs (antioxidation, immunomodulation, anti-inflammation, antiangiogenesis, and antiproliferation) are dependent on absorption and systemic bioavailability. Thus, small-molecule PCs with high absorption are particularly promising agents for clinical applications [37].

The antioxidative properties of PCs have been demonstrated in multiple OS-associated diseases [38]. Studies conducted* in vitro *and* in vivo* have demonstrated that PCs can effectively resist OS-induced damage and augment cellular antioxidant capacity by direct molecular scavenging and by modulation of various downstream signaling pathways associated with stress responses. Several researchers found that PCs contributed to the prevention of UV-induced skin disorders, diabetic retina injury, or zearalenone-induced liver damage by activating Nrf2 pathway or inhibiting MAPK/NF-*κ*B pathway, by scavenging hydroxyl radicals and superoxide anions, and by upregulating endogenous antioxidants and detoxication enzymes, such as hemeoxygenase-1 (HO-1), catalase (CAT), superoxide dismutase (SOD), and glutathione peroxidase (GSH-Px) [39–42]. In addition, Yamakoshi et al. demonstrated that PCs have low toxicity and are safe for dietary administration in rats [43, 44]. Fujii et al. further reported that the PCs constituent Oligonol, found in lychee extract, is safe, nontoxic, and nonmutagenic to healthy humans even at 200 mg/day for three months [45]. Meanwhile, some clinical trials of PCs have been performed for the assessment of safety and the treatment of clinic diseases in healthy subjects and pregnant women, which have demonstrated that daily oral intake of PCs up to 2500 mg is safe in healthy adults and PCs serve as a safe and effective therapy for pregnant women with condyloma acuminata [46, 47]. These results indicate that PCs are safe antioxidants with no apparent side effects and so may be widely utilized in clinical medicine and cosmetology. However, more systematic toxicity tests on PCs are needed to investigate the safety of other constituents and to evaluate their effectiveness in the food ecosystem.

## 3. Mechanisms against OS

OS is essentially a state of redox imbalance originating from relative overproduction of ROS/RNS or decreased antioxidant capacity as indicated by the reduced oxidized glutathione (GSH/GSSG) or NADPH/NADP^+^ ratio. OS can result from the overproduction and accumulation of prooxidants such as ROS, lipid peroxides, NO, and superoxide radical (O_2_^−^) or from the relative insufficiency of antioxidant molecules (GSH) and enzymes such as CAT, SOD, and GSH-Px [39]. As the second messenger or signaling molecule, ROS play a key role in the initiation and development of OS [48, 49]. ROS at low levels are beneficial to cells and tissues (e.g., cell proliferation, tissue repair, and angiogenesis) [50–52]; conversely, at high levels ROS contribute to cell damage, apoptosis, and/or death [53, 54]. Normally, ROS are rapidly detoxified by endogenous antioxidants; however, accumulated ROS can initiate a surge of toxic biochemical reactions that result in direct damage to DNA, lipids, and proteins [55, 56]. Increasing evidence has indicated that ROS have the ability to mediate various signaling pathways (e.g., Nrf2, MAPK, NF-*κ*B, and PI3K/Akt), ion channels, and transporters and modify protein kinase and ubiquitination/proteasome system [57, 58]. Although the knowledge of ROS-mediating mechanism is known well, further studies on ROS interacting with signaling pathways are still required. There is now compelling evidence implicating OS and associated signaling pathways in cardiovascular, metabolic, neurodegenerative, and inflammatory diseases, as well as immune diseases [59]. Furthermore, there is also rapidly accumulating evidence that PCs can prevent OS damage by downregulating these same molecular species and signaling pathways [12].

### 3.1. Repair of DNA Damage

Excessive ROS production during OS can directly induce DNA damage or mutations. For instance, ROS produced by photo-OS reacts with nucleobases or the 2-deoxyribose moiety and results in nuclear DNA oxidative damage through single pyrimidine or purine modification, interchain cross-links, DNA-protein adduct formation, and apurinic/apyrimidinic (AP) site formation. Exposure to UV can induce the formation of highly mutagenic cyclobutane pyrimidine dimers (CPDs) and other free radicals potentially damaging to genomic DNA [60, 61]. Mitochondrial DNA (mtDNA) is even more susceptible to ROS-mediate mutagenesis and destruction than nuclear DNA due to its proximity to the electron transport chain, a major site of ROS generation, and lack of protective histones [62, 63]. Primary damage to nuclear DNA and mtDNA facilitates additional mutations, which may ultimately result in chronic diseases and cancers.

PCs have been shown to prevent OS-induced DNA damage and promote DNA repair through the following pathways [64–67]: (1) scavenging oxidative species (e.g., ROS and RNS) and free radicals, thereby disrupting direct OS damage and redox chain reactions; (2) enhancing the functions of DNA repair enzymes; (3) dose-dependently inhibiting CPD formation; (4) rapidly repairing CPDs through the induction of IL-12; (5) promoting the nucleotide excision repair mechanism; and (6) inhibiting DNA hypomethylation.

### 3.2. Prevention of Lipid Peroxidation

OS-related products attack multiple biomolecules; particularly polyunsaturated fatty acids (PUFAs) are vulnerable, which can result in membrane injury through inactivation of membrane receptors and enzymes, reduced membrane fluidity, increased membrane permeability to ions, and in extreme cases cell membrane rupture and release of organelles [68, 69]. Excessive ROS not only directly damage PUFAs but also initiate a self-perpetuating chain reaction in which lipid peroxides as well as unstable FA radicals are generated and rapidly broken down to form additional FA radicals [70–72]. PCs can stabilize and inactivate free radicals by donating an electron to free radical -OH groups attached to the phenolic ring, which helps to terminate oxidative chain reactions. Indeed, Mittal et al. found that PC treatment substantially inhibited UVB-induced lipid peroxidation [69, 73]. Moreover, PCs combined with DHA-OR could improve PUFAs, ameliorate lipid hydroperoxides, and increase the detoxification of postprandial xenobiotics in rats liver [74]. In addition, PCs protected against cadmium-induced ROS production, free radical production, and lipid peroxidation in rat erythrocytes and lymphocytes [75].

### 3.3. Modulation of Signaling Pathways Involved in OS

In addition to oxidation of macromolecules, ROS generated during OS may act as second messengers to activate or inhibit signal pathways that control the expression of downstream stress-responsive genes. Disruption or aberrant activation of these pathways can lead to premature aging, inflammation, and oncogenesis [59]. In the following section, the effects of PCs on OS-related signaling pathways are discussed.

#### 3.3.1. Inhibition of MAPK Pathways

The MAPK family kinases p38 MAPKs, c-Jun amino-terminal kinases (JNKs), and extracellular signal-regulated kinases (ERKs) are important regulators of transcriptional cascades mediating stress responses in cells [76, 77], including responses to UV, heat, and toxic chemicals [78]. OS triggers the activation of JNKs, p38 MAPKs, and ERKs through ROS-induced enhancement of phosphorylation, resulting in nuclear translocation and promoting expression of stress-response factors related to OS, cell proliferation/apoptosis, inflammation, and tissue remodeling (such as vascular growth) [79–81]. However, PCs can directly downregulate stress-activated MAPK pathway activities (e.g., IL-17-stimulated ERK, p38, and JNK activities) resulting in suppression of ROS production, OS damage, and apoptosis-related pathways [82]. For instance, PCs not only diminished ethanol-induced ROS generation but also enhanced the expression and activity of antioxidant enzymes via ERK, JNK, and p38 MAPK pathways in both cultured cells and rat liver [83]. In addition, PCs enhanced Nrf2 expression and activated Nfr2 antioxidant response element- (ARE-) mediated transcription via p38 MAPK and PI3K/Akt pathways in HepG2 cells, thereby increasing phase II/antioxidant enzyme expression [84].

#### 3.3.2. Suppression of the NF-*κ*B Pathway

Transcription factor NF-*κ*B is activated by multiple cellular stressors (including oxidants and antigens) and in turn regulates the expression of many genes involved in OS, apoptosis, and inflammation [85]. Its target genes mainly encode regulators of the immune/inflammatory response, such as immune receptors (IFN-*γ* receptor, MHC, IL-2 receptor), cytokines (TNF-*α*, IL-6, IL-1), adhesion molecules (VCAM-1, ICAM-1), prooxidant enzymes (COX-2, iNOS), and chemokines (MCP-1, MIP-1*α*, IL-8). NF-*κ*B also enhances the transcription of antioxidant enzymes and antiapoptotic proteins [86]. Normally, NF-*κ*B proteins are retained in the cytoplasm through interactions with NF-*κ*B inhibitory proteins (I*κ*Bs); under OS, however, I*κ*Bs are phosphorylated, dissociated from NF-*κ*B, and degraded, allowing NF-*κ*B translocation into the nucleus [87]. MAPKs signals are important upstream regulators of the NF-*κ*B pathway [88]. PCs can reduce OS damage and inflammation by suppressing NF-*κ*B, ERK, p38, and JNK phosphorylation. Via blocking NF-*κ*B and MAPK pathways, PCs in turn inhibit the mRNA expression of proinflammatory cytokines like TNF-*α* and IL-1*β* as well as the inflammatory prostaglandin products of COX-2 [89, 90]. In addition, PCs can suppress the replication of respiratory syncytial virus (RSV) by blocking RSV-induced NF-*κ*B, p38 MAPK/JNK, AP-1, and ERK activities [91].

#### 3.3.3. Activation of Nrf2 Pathways

Nrf2 is a crucial transcriptional regulator in OS and facilitates the expression of cytoprotective genes in response to OS. Under normal conditions, Nrf2 remains in an inactive cytoplasmic form through binding to Kelch-like ECH associating protein 1 (Keap1), which facilitates Nrf2 degradation [92]. OS triggers Nrf2−Keap1 dissociation and Nrf2 nuclear translocation, where it binds to the AREs and promotes activation of various antioxidant enzymes/proteins [93–95] such as HO-1, phase II detoxification enzymes peroxiredoxin 1, glutamate-cysteine ligase catalytic subunit, and NAD(P)H quinone dehydrogenase 1 (NQO1) [96, 97]. PCs can induce Nrf2 expression and ARE-mediated transcription [84], thereby reducing OS. For instance, PCs inhibited lead-induced liver OS damage and elevated antioxidant capacity via activation of Nrf2/ARE signaling [98]. Oligomeric PCs also markedly enhanced the nuclear translocation of Nrf2, promoted the expression of HO-1, NQO1 and thioredoxin reductase 1, and suppressed H_2_O_2_-induced OS damage in A549 cells [99].

#### 3.3.4. Regulation of Other Signaling Pathways

The Janus kinase-signal transducer and activator of transcription (JAK/STAT) pathway is another important regulator of inflammatory factors and cytokines associated with OS. The JAK/STAT pathway is activated by ROS and hypoxia/reperfusion or osmotic stress through binding of induced cytokines to their specific receptors [100, 101]. The PI3K/Akt pathway also regulates OS and cell proliferation, and abnormalities in this pathway have been implicated in the initiation and progression of cancer [102]. PCs may prevent apoptosis and alleviate neurological impairment in type 2 diabetes (T2D) model Sprague−Dawley rats with focal cerebral ischemia by decreasing STAT1 expression and inhibiting JAK/STAT signaling [103]. PCs also inhibited high glucose-induced vascular smooth muscle cell growth by blocking the PI3k/Akt-dependent signaling pathway and ROS overproduction [104].

**Figure 2:**
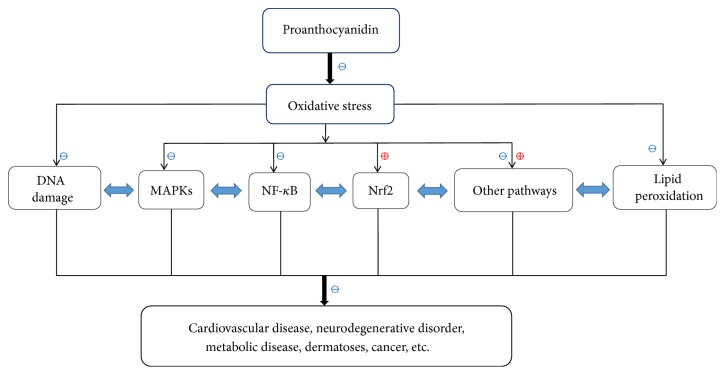
*PCs control OS-related diseases via mediating several molecular targets and signaling pathways*. PCs effectively suppress OS through repairing DNA damage, preventing lipid peroxidation, and modulating signaling pathways (e.g., Nrf2, MAPKs, NF-kB pathways) and, further, treat OS-related diseases like cardiovascular diseases, neurodegenerative disorders, metabolic diseases, skin disorders, and cancer. ⊕ means “to promote or enhance”; ⊖ means “to inhibit or suppress.”

The many pathways and factors involved in OS seldom work in isolation. For example, STAT1 can be directly phosphorylated by p38 MAPK* in vitro*, which implies that MAPKs and STAT are convergent pathways regulating OS [105]. Similarly, PI3K and MAPK pathways influence Nrf2 transcription and phase II detoxifying/antioxidant enzymes expression [106, 107]. Therefore, PCs may alleviate and control OS by mediating broad OS-associated pathways via targeting specific signaling nodes (Figure 2). Further, individual structurally distinct PCs may exert diverse therapeutic effects depending on structure. This property may enable the same PCs extract to suppress multiple etiologically distinct OS-related clinical disorders.

## 4. Clinical Applications of PCs for OS-Related Diseases

Clinical and experimental evidence implicate that OS is in numerous acute/chronic diseases including cardiovascular, neurodegenerative, metabolic, and inflammatory disorders [59]. Owing to antioxidant efficacy, PCs may be powerful clinical tools for the management of these diseases.

### 4.1. Cardiovascular Diseases

There is substantial evidence for PC efficacy against cardiovascular diseases. OS is believed to be an essential mechanism in the pathogenesis of cardiovascular diseases and vasculopathies such as atherosclerosis, hypertension, heart failure, and restenosis after angioplasty [9, 108]. ROS disrupt myocardial calcium homeostasis, which can lead to arrhythmia and cardiac remodeling by aberrant induction of various signaling pathways and gene expression cascades [109]. ROS-mediated lipid peroxidation contributes to the initiation and progression of atherosclerosis [110]. Deoxycorticosterone acetate- (DOCA-) salt promotes the development of hypertension, cardiovascular remodeling, and cardiovascular dysfunction by increasing p38 MAPK and JNK1/2 phosphorylation. On the contrary, PCs can reduce high blood pressure induced by DOCA-salt and ameliorate cardiovascular remodeling by suppressing ROS generation and p38MAPK pathway activation [111]. Simultaneously, PCs are able to reduce risk factors for cardiovascular diseases by diminishing lipid peroxidation, decreasing blood pressure, and improving hypertriglyceridemia [112].

### 4.2. Neurodegenerative Disorders

It has been confirmed that PCs provide neuronal protection against degenerative diseases by scavenging ROS [113]. OS is a primary pathogenic mechanism in multiple neurodegenerative diseases, such as Parkinson's disease (PD), amyotrophic lateral sclerosis, Alzheimer's disease (AD), and epilepsy [114]. Lipid peroxidation contributes to a chain reaction implicated in neurotoxicity and neuroinflammation. PUFAs are abundant in the nervous system and prefer to present in neuronal membranes than other cellular membranes [115]. Furthermore, certain species such as the *ω*-3 LC-PUFA docosahexaenoic acid (DHA) is unique to the nervous system and exquisitely sensitive to OS [116]. The PI3K/Akt signaling pathway plays a protective role in neurodegenerative diseases [117], and PCs can disrupt lipid peroxidation chain reactions to protect neurons through PI3K/Akt signaling pathway modulation [69, 73]. For instance, PCs protected against cadmium-induced oxidative neurotoxicity in mouse brain by activating Akt phosphorylation and preventing lipid peroxidation-dependent membrane damage [118]. Also, PCs offered powerful protection against OS-related DNA damage and lipid peroxidation in AD and PD models [119]. PCs, meanwhile, attenuated neurotoxicity and alleviated neurodegeneration in PD cell models via control of OS progression and preservation of mitochondrial function [120].

### 4.3. Metabolic Diseases

Metabolic disorders such as obesity, insulin resistance, T2D, hepatic steatosis, and hyperlipidemia are accompanied by ROS overproduction, lipid peroxidation, and alterations in MAPK and JNK signaling [121, 122]. Patients with metabolic disorders like T2D often have abnormal serum markers of OS [123]. In cell-based T2D models, elevated glucose-induced ROS accumulation and OS result in injury to cardiomyocytes, endothelial cells, and neurons [124, 125]. Therefore, treatments against OS are indispensable for metabolic diseases. It has been verified that PCs are effective for ameliorating T2D-produced damage of various tissues by increasing pancreatic GSH and reducing lipid peroxidation as well as total nitrate/nitrite levels [126]. Dietary supplementation of PCs dose-dependently prevented the development of hyperglycemia in diabetic obese mice, which suggests that PCs may help control the onset of T2D [127]. Moreover, the levels of total cholesterol, triacylglycerol (TG), low density lipoprotein-cholesterol (LDL-C), glutamate pyruvate transaminase, glutamic-oxal(o)acetic transaminase (GOT), and MDA were significantly reduced while SOD and GSH-Px activities were enhanced in a PC-treated liver injury model. This suggests that PCs may protect against high-fat diet-induced fatty liver disease [128].

### 4.4. Dermatoses

A redox imbalance in the cutaneous microenvironment offers favorable conditions for the initiation and development of skin diseases. Numerous studies have shown that OS contributes to the occurrence and progression of skin photoaging, skin cancer, chloasma, vitiligo, skin trauma, polymorphous light eruption, psoriasis, alopecia areata, atopic dermatitis, and allergic purpura [129–136]. OS can initiate and aggravate dermatoses attacks by damaging DNA, lipids, and proteins, overactivating cytokines/immune mediators, and modifying the activities of Nrf2, MAPK, NF-*κ*B/p65, and PI3K/Akt signaling pathways. As antioxidants, PCs are effective for treating dermatoses by repairing damaged DNA, lipids, and proteins. In addition, PCs control cytokine/immune mediator release and regulate OS-related signaling pathways. For instance, PCs attenuated UV-induced OS-mediated dermatoses in human skin through suppression of UV-induced OS and activation of NF-*κ*B and MAPK pathways [40].

### 4.5. Cancer

OS may cause epigenetic perturbations and mutations by modifying chromatin proteins and damaging DNA [137]. ROS are critical mediators of growth factor receptor signaling and actively participate in cancer cell proliferation [138, 139]. Cancer cells always exhibit accelerated metabolism, and high-level ROS generation is required to maintain their strong proliferation potential. Therefore, targeted regulation of ROS is a promising strategy for cancer therapy [140]. It has been demonstrated that PCs can inhibit proliferation of oral squamous cell carcinoma in a dose-dependent manner and reduce the proliferation of cervical cancer cell lines [141]. Moreover, dietary PCs suppressed UVB-induced cutaneous cancer development by promoting the repair of damaged DNA via nuclear excision repair mechanisms and by stimulating DNA repair-dependent immune activity via effector T cells and dendritic cells [26]. These results underscore the promise of PCs as candidate drugs for the treatment and prevention of cancer.

In addition, OS and related signaling pathways are implicated in numerous additional immune system disorders (e.g., systemic lupus erythematosus [142] and Behcet's disease [143]), urinary system disorders (e.g., uremia) [144], and digestive system diseases (e.g., irritable bowel disease [145]). Further, these disorders may also involve molecules and pathways targeted by PCs, including caspases, PERK/NRF2, NADPH oxidase 4, and JNK/MAPK signaling cascades [146].

## 5. Conclusions

In summary, PCs are natural phytocompounds with potential health benefits, including antioxidant, antimutagenic, antineoplastic, anticytotoxic, and anti-inflammatory activities. Therefore, these agents may be useful for the prevention and treatment of OS-related cardiovascular, neurodegenerative, metabolic, and inflammatory diseases as well as various cancers. Furthermore, compared with synthetic compounds, PCs are readily affordable without side effects. In this review, we summarized the molecular mechanisms and signaling pathways involved in OS, which can be considered as potential targets of PCs treatment. However, more studies about the safety and the effectiveness of different constituents of PCs are needed. Besides, large-sample clinical trials are required. We hope that this review enhances appreciation of the therapeutic potential of PCs against OS-related diseases and that these perspectives facilitate the development of novel PCs-based therapeutic agents.

## Figures and Tables

**Table 1:** Primary distribution of PCs in various plants [27].

Fruit/seeds/peel	*Grape, Brassica campestris, Camellia oleifera, Glycine max *var.*, Oryza, Sorghum vulgare, Theobroma cacao, Vicia faba, Lablab purpureus, Litchi chinensis, Hippophae rhamnoides, Malus pumila, Rubus allegheniensis, Fragaria vesca, Fructus crataegi, Dimocarpus longan, Vaccinium myrtillus, Elaeagnus angustifolia, Oxycoccus, Diospyros kaki, Garcinia mangostana, Punica granatum *

Leaves	*Fructus crataegi, Leucaena leucocephala *cv.*, Reyan *No.1*, Ginkgo biloba, Betula platyphylla, Vitis *spp.*, Thea viridis, Scrophularia ningpoensis, Hypericum perforatum, Laurus nobilis, Eucalyptus *spp.

Flower	*Fructus crataegi, Rosa rugosa, Dimocarpus longan, Pharhiris nil qianniuhua, Ipomoea aquatica, Trifolium pratense, Nymphaea tetragona*

Root/stem	*Sanguisorba officinalis, Pueraria lobata, Rheum palmatum, Anisophyllea dichostyla, Ipomoea batatas*

## Supplementary Materials

Supplementary Materials The graphical abstract presents a remarkable snapshot of PCs characteristics and its role in mediating oxidative stress as well oxidative stress-associated disorders via regulating specific molecules and signaling pathways.
