# Supplementary figures and images for: Proanthocyanidins against Oxidative Stress: From Molecular Mechanisms to Clinical Applications
Source: Biomed Res Int. 2018 Mar 12;2018:8584136. doi: 10.1155/2018/8584136 (PMC5884402; doi:10.1155/2018/8584136)

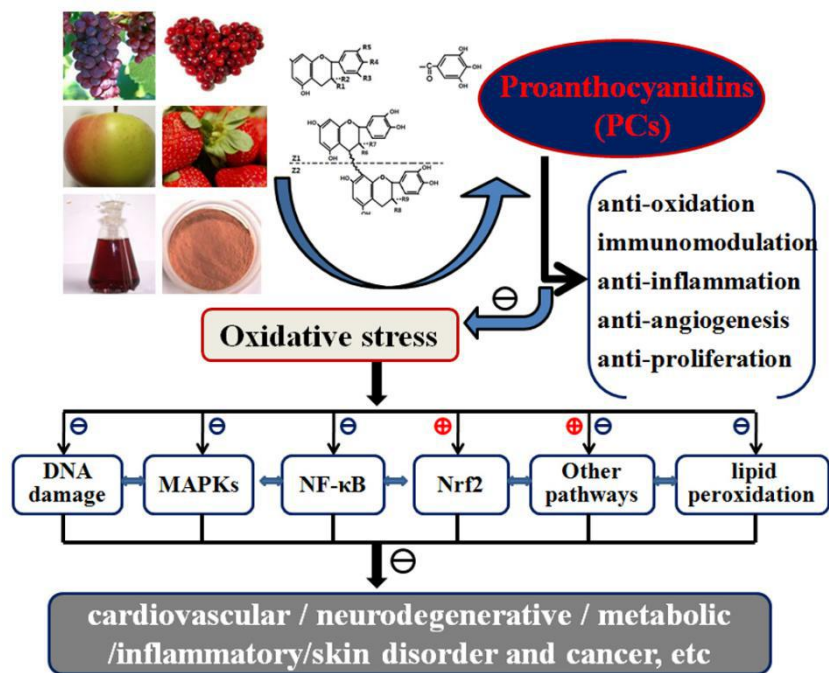

Graphical Abstract

Supplement: Supplementary Materials — The graphical abstract presents a remarkable snapshot of PCs characteristics and its role in mediating oxidative stress as well oxidative stress-associated disorders via regulating specific molecules and signaling pathways. [file 8584136.f1.pdf]
